# Understanding epigenetic changes in aging stem cells – a computational model approach

**DOI:** 10.1111/acel.12177

**Published:** 2014-01-15

**Authors:** Jens Przybilla, Thimo Rohlf, Markus Loeffler, Joerg Galle

**Affiliations:** 1 Interdisciplinary Center for Bioinformatics, University LeipzigHaertelstr. 16-18, 04107, Leipzig, Germany; 2 Institute for Medical Informatics, Statistics and Epidemiology, University LeipzigHaertelstr. 16-18, 04107, Leipzig, Germany; 3 Max-Planck-Institute for Mathematics in the SciencesInselstr. 22, 04103, Leipzig, Germany

**Keywords:** aging of stem cells, clonal competition, DNA methylation, histone modification, mathematical model, population dynamics

## Abstract

During aging, a decline in stem cell function is observed in many tissues. This decline is accompanied by complex changes of the chromatin structure among them changes in histone modifications and DNA methylation which both affect transcription of a tissue-specific subset of genes. A mechanistic understanding of these age-associated processes, their interrelations and environmental dependence is currently lacking. Here, we discuss related questions on the molecular, cellular, and population level. We combine an individual cell-based model of stem cell populations with a model of epigenetic regulation of transcription. The novel model enables to simulate age-related changes of trimethylation of lysine 4 at histone H3 and of DNA methylation. These changes entail expression changes of genes that induce age-related phenotypes (ARPs) of cells. We compare age-related changes of regulatory states in quiescent stem cells occupying a niche with those observed in proliferating cells. Moreover, we analyze the impact of the activity of the involved epigenetic modifiers on these changes. We find that epigenetic aging strongly affects stem cell heterogeneity and that homing at stem cell niches retards epigenetic aging. Our model provides a mechanistic explanation how increased stem cell proliferation can lead to progeroid phenotypes. Adapting our model to properties observed for aged hematopoietic stem cell (HSC) clones, we predict that the hematopoietic ARP activates young HSCs and thereby retards aging of the entire HSC population. In addition, our model suggests that the experimentally observed high interindividual variance in HSC numbers originates in a variance of histone methyltransferase activity.

## Introduction

Epigenetics is commonly described as the study of heritable changes in gene expression or cellular phenotype caused by mechanisms other than changes in the underlying DNA sequence (Holliday, 2006). Epigenetic mechanisms are key players controlling development and cell differentiation (Bibikova *et al*., 2008; Cedar & Bergman, 2011). However, once a tissue has been specified, it is commonly expected that defined chromatin features have to be conserved throughout life to ensure tissue function. In sharp contrast, progressive alterations of the epigenome have been observed during aging. In the following, we call them ‘epigenetic drifts’. These drifts have been demonstrated in many eukaryotes including mice and human (Berdasco & Esteller, 2012). They appear to be tissue- and species-specific (Han & Brunet, 2012) and comprise changes of DNA methylation states as well as of histone modifications; both capable of affecting the transcriptome of the cell.

Here, we focus on the interplay between the regulatory mechanisms underlying these changes. We introduce a computational model of these mechanisms to provide a mechanistic understanding of age-related epigenetic drifts. Moreover, we aim at linking these mechanisms to phenotypic changes of cells in order to derive hypotheses on the emergence of age-related phenotypes (ARPs) on the population level. For this purpose, we combine our model of transcriptional regulation with an individual cell-based model of stem cell populations, which allows us to simulate aging on the molecular, cellular, and population level. We hypothesize that ARPs are a consequence of epigenetic drifts, which originate in the limited cellular capability to inherit epigenetic information.

### Inheritance of histone states

In the last decade, an universe of possible histone modifications has been described; among them ubiquitylation, phosphorylation, acetylation, and methylation (Tollervey & Lunyak, 2012). These modifications are reversibly catalyzed by specific enzymes. Thus, they underlie fluctuations on different time scales (Barth & Imhof, 2010) ranging from highly dynamic behavior in the case of acetylation (half-life time in the range of minutes) to the more stable methylation states of extended chromatin regions (half-life time in the range of days). The latter have been suggested to become stable due to interaction between cooperative nucleosomes that carry the same modification (Dodd *et al*., 2007; Binder *et al*., 2013). Nonetheless, large fluctuations can also induce spontaneous (de-) methylation of all histones in such cooperative units. A particular source of such fluctuations in modifications of the histones H3 and H4 is cell replication. During cell division, intact H3–H4 tetramers of the mother strand are randomly distributed onto the daughter strands. They form the core of the new nucleosomes that are complemented by de novo synthesized ones. This leads to a dilution of the modified nucleosomes at the daughter strands that can induce further de-modification (Binder *et al*., 2013). This kind of limited inheritance could be avoided if identical H3 and H4 sister histones would become split and copied during cell division (Margueron & Reinberg, 2010). This scenario, however, is not compatible with recent observations of a large portion of asymmetric nucleosomes (Voigt *et al*., 2012) and with the small rates of splitting of H3–H4 tetrameres during cell division (Xu *et al*., 2010).

### Inheritance of DNA methylation

Compared with histone modifications, DNA methylation is much more stable. However, age-related changes in DNA methylation, namely local promoter hyper-methylation and global hypo-methylation, have been recognized more than 10 years ago (Dunn, 2003). Recently, it has been demonstrated that progressive age-dependent DNA methylation changes start already before adulthood (Takasugi, 2011). The origin of these changes is not fully understood so far. We suggested age-related changes of the histone modification state as a possible source (Przybilla *et al*., 2012).

In principle, DNA methylation states are inherited during replication by the action of maintenance and changed by de novo methyltransferases. The resulting stationary state depends on the activity of these methyltransferases and in particular on their recruitment. Histone modifications have been involved in this recruitment. Actually, both DNA methyltransferase recruiting and repelling action of histone modifications have been described by Cedar & Bergman (2009). A prominent example of such crosstalk is that between methylation at lysine 4 of histone H3 (H3K4) and the de novo DNA methylation machinery. In ‘young’ cells, many genes with CpG-rich promotors are associated with nucleosomes that are tri-methylated at H3K4 (H3K4me3). This modification, known to be associated with active transcription, protects the associated DNA from becoming methylated by the de novo methyltransferase DNMT3a (Ooi *et al*., 2007). Spontaneous de-modification of the histones during aging enables the DNMT3a to become active. We suggested that this represents a basic mechanism of age-related hyper-methylation (Przybilla *et al*., 2012). Indeed, loss of H3K4me3 has been observed during aging in human and mice (Cheung *et al*., 2010; Kuzumaki *et al*., 2010). Thus, although also other histone modifications, as e.g., H3K9me3, have been implicated in controlling DNA methylation (Cedar & Bergman, 2009), we here focus on the interplay between H3K4me3 and the DNA methylation machinery.

### Age-related phenotypes

The particular mechanisms how histone modification and DNA methylation affect transcriptional activity of genes are still under debate. However, their crucial role regarding age-related transcriptional changes is commonly accepted (Pollina & Brunet, 2011). In stem cells, these changes have been demonstrated to be associated with a functional decline including a reduced regeneration potential and a deregulation of the stem cell pool.

Age-related phenotypes are probably documented best for hematopoietic stem cells [HSCs, (Geiger *et al*., 2013)]. In the hematopoietic system, the number of stem cells increases with age (Dykstra *et al*., 2011). Clones of young and aged cells coexist, but the aged ones do less efficiently contribute to differentiated progeny (Verovskaya *et al*., 2013). One objective of our study is to provide an explanation of these experimental findings in HSCs.

In the following, we first give a short description of our computational model. Afterward, we provide simulation results on different model scenarios and discuss our findings.

## Results

### Basic model

**Figure 1:**
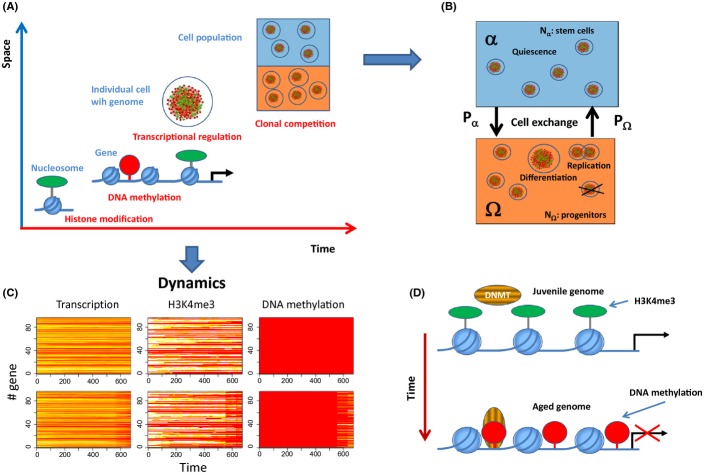
Multi-scale model of epigenetic drifts in aging cells. (A) Time and length scales covered by our model. The basic unit of the model is the nucleosome, the histones of which undergo H3K4 (de-) modification. The average H3K4me3 level controls the access of CpG-methylating enzymes to nearby CpG sites. Both H3K4 and DNA methylation interfere with the transcription factor-network of the cell that is defined by its genome. All cells are equipped with the same genome but underlie individual aging and thus reside in individual regulatory states. Aging depends on signals provided by the environment. We consider two environments α and Ω. (B) In the α environment mimicking a stem cell niche (blue) cells are quiescent, while in the Ω environment (orange), they can proliferate and differentiate with rate *R* and *q*, respectively. Differentiated cells are removed from the system. Cells make transitions between α and Ω with probabilities P_α_ and P_Ω_. (C) Regulatory profiles of individual cells: Transcription, H3K4me3 modification, and DNA-methylation profiles (red-yellow-white: low–high values) for a cell fixed in environment α (upper row) and Ω (lower row). H3K4me3 modification and DNA methylation are given as the fraction of modified nucleosomes and methylated CpG sites associated with the gene, respectively. Time is given in computational time steps Δt. Proliferation is required for changes in DNA methylation, while changes of H3K4me3 modification occur also in quiescent cells. (D) Age-dependent phenotypic changes. Initially the nucleosomes carry H3K4me3 modifications which protect the associated DNA from becoming methylated. Over time this modification is lost enabling methylation of the nearby CpG sites and stable silencing of the associated gene.

To study processes of stem cell aging *in silico*, we apply a multiscale model of transcriptional regulation recently developed by us (Przybilla *et al*., 2012). The time and lengths scales covered by the model are explained in Figure 1A. The model describes transcriptional regulation within a cell carrying an artificial genome (see Supporting Information), the genes of which are part of a cis-regulatory network. Interaction between two genes of this network is described by means of sequence specific binding of the transcription factor (TF) encoded by one gene and interaction of this TF with polymerase II bound to the promoter of the other gene and *vice versa* (Binder *et al*., 2010). These interactions represent a first regulatory layer of gene expression in the model. Two further layers are represented by mechanisms of chromatin reorganization; namely H3K4 trimethylation and DNA methylation. Histone modification is described as a balance between permanent modification and de-modification reactions of the histones of cooperatively acting nucleosomes (Binder *et al*., 2013). Due to positive feedback mechanisms involved in the recruitment of histone modifying complexes, high- and low-modification states can be stable at the same time, that is, the system can be ‘bistable.’ Thereby, bistability depends on the number of cooperatively acting nucleosomes and on the methylation state of the associated DNA. The latter can change in the model during cell replication only due to limited maintenance and potential de novo methylation (Sontag *et al*., 2006; Przybilla *et al*., 2012). The recruitment of the DNA methylation machinery is prevented if the associated nucleosomes are H3K4me3 modified. Changes of the H3K4me3 state impact transcription of associated genes by changing their promoter activity, while DNA methylation impacts transcription only indirectly by changing the histone modification probability. In case, the expression of genes is controlled by bistable H3K4me3 states of associated nucleosomes, we call them in short ‘bistable genes.’ Details about the model can be found in the ‘Material and Methods’ section.

### Systems dynamics

We consider two different environments the cells can reside in (Roeder & Loeffler, 2002; Glauche *et al*., 2011); a niche environment α where cells are quiescent, that is, have zero proliferation rate (*R* = 0) and an environment Ω where proliferation is active (*R* > 0). Cells can change between these two environments with probabilities P_α_ and P_Ω_ for a switch from α to Ω and from Ω to α, respectively. Moreover, cells in Ω differentiate with rate *q* and are removed from the system (Fig. 1B). In our simulations, cells do not interact, that is, they behave independently. Each cell is characterized by its specific time-dependent transcriptional, H3K4me3 modification and DNA methylation profile. We assumed that in the initial state of the system all histones are modified and all CpGs are un-methylated. The initial transcription state of all genes is determined by these conditions. Figure 1C shows the behavior of two cells; one fixed in the α- and one in the Ω-environment. For the cell behavior, two different time scales are important. The first one is the time scale of fluctuations of the modification of individual histones (short time scale < 1 h (Hayashi-Takanaka *et al*., 2011)) which is determined by the histone (de-) modification rates k_M_, and k_D_ for modification and de-modification, respectively. The second one is the time scale of cell division (long time scale > 10 h, typically for mammalian cells during homeostasis) characterized by the replication rate *R*.

### The cell states evolve as following:

- On short time scales the stochastic histone (de-) modification dynamics drives the system toward a quasi steady state. The related fluctuations of the modification level are normally small. Thus, genes associated with high H3K4me3 levels are stably transcribed.
- On longer time scales, the cells replicate depending on the environment. Cell division depends on a stochastic growth process of the cell volume (Galle *et al*., 2005). It takes place if a threshold cell volume is reached. During division, the histones of the mother cell are randomly distributed onto the daughter cells. As a consequence, H3K4me3-modified nucleosomes become diluted. This loss enables DNA methylation of nearby CpG sites. DNA methylation in turn stabilizes the unmodified H3K4 states and results in stable gene silencing.

In the following, we consider these *drifts of the cells regulatory states on long time scales as aging*. They convert active genes into silenced genes (Fig. 1D). We expect related changes to contribute to the development of age-related phenotypes on the level of cells, tissues, and organisms.

### Age-related phenotypes

In the following, we assume that ARPs on the cellular level are induced by a defined expression level of selected genes. These genes have to be considered as context specific as they depend on particular epigenetic state of the tissue. This is in agreement with experimental findings that age-related expression changes do rarely overlap between different tissues and/or species (de Magalhaes *et al*., 2009). All parameters controlling H3K4me3 and DNA methylation dynamics are assumed to be constant in a particular simulation. In the following, we present simulation results according to different aging scenarios. Population heterogeneity is quantified in terms of the variance of the regulatory profiles within the individual cells. Clonal development in the cell population is analyzed monitoring the number of clones, the size of the individual clones and the distribution of ARPs within the clones.

### Epigenetic drifts affect stem cell heterogeneity

**Figure 2:**
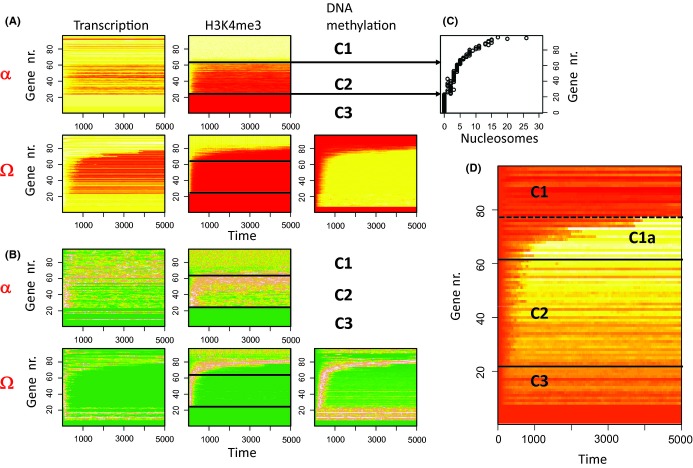
Simulation results for an age-independent phenotype: (A) Regulatory states for each gene averaged separately over all cells in the α- and Ω-environment (red-yellow-white: low-high, 200 Δt ~ 1 generation). Genes are sorted by the average H3K4me3 modification in Ω. Values for transcription and H3K4me3 modification are normalized to their maximum value. H3K4me3 and DNA-methylation are given as the fraction of modified nucleosomes and methylated CpG sites associated with the gene, respectively. Three different sets of genes C1–C3 can be defined (see text). H3K4me3 decreases for C2-genes in both environments and for C1a-genes in Ω. In Ω cell proliferation allows for increasing DNA methylation, while DNA of quiescent cells in α remains un-methylated (not shown). (B) Variance of the states for each gene averaged separately over all cells in the α- and Ω-environment. (green-brown-white: low–high, all values normalized to their maximum value). Genes sorted as in (A). Regulatory states of C2-genes, in particular H3K4me3 states, are more heterogeneous in α compared with Ω. (C) Number of cooperative nucleosomes associated with the genes. (D) Differential expression of the genes in the two environments (lnα–lnΩ). The genes of set C2 and of set C1a show higher expression (yellow, white) in the niche.

In a first setting, we assumed that all parameters of the model are age-independent (no ARP). Figure 2 summarizes simulation results obtained for these conditions assuming no exchange between α and Ω (P_α_ = P_Ω_ = 0). In the niche environment α, aging cannot occur as the cells remain quiescent, while in the Ω-environment, long-term drifts are detectable (Fig. 2A). According to their regulatory states, three different sets of genes can be identified: C1-genes are associated with nucleosomes that are stably modified in cells residing in the niche. In Ω, some of them lose H3K4me3 modification. These genes define the subset C1a that acquires DNA methylation and becomes silenced. Remarkably, C1a-genes are bistable, that is, the associated nucleosomes can in principle regain histone modification in course of stochastic modification reactions (see: Fig. S1). Thus, their silencing can be reversed. C2-genes are associated with nucleosomes that undergo fluctuations of their modification level in α, while completely losing modification in Ω. Accordingly histone modification and transcriptional states of C2-genes are much more heterogeneous in cells within α compared to cells within Ω (Fig. 2B). In contrast to C1a-genes, silencing of C2-genes is not reversible as their states are not bistable. C3-genes are shorter than 200 bases and thus are not associated with nucleosomes. A subgroup of them also does not contain CpGs. We assigned them zero histone/DNA methylation level, respectively.

Our simulation results show that cell proliferation impacts not only the regulatory states but also their variance. Reversible state changes in the niche are associated with high variance of transcription and histone modification of C2-genes, while gene silencing during aging is associated with variance in DNA methylation in C1a-genes. Figure 2D shows that both gene sets show higher expression in the α-environment compared with the Ω-environment. Irreversible silencing of C2-genes occurs after a few cell cycles. Thus, it is indicative of stem cell expansion. Accordingly, C2-genes may be considered as ‘stem cell markers.’ In contrast, reversible silencing of C1a-genes requires many cell cycles. These genes can be considered as ‘aging marker.’

Which genes actually refer to which set depends on the particular choice of the parameters of our model, as for example the histone (de-)modification constants, and thus, maybe considered as ‘tissue’-specific. Under the chosen conditions, the differences between the genes are dominated by the number of cooperatively acting nucleosomes associated with the genes (Fig. S1). Figure 2C demonstrates that accordingly, the genes sets can be classified by the number of these nucleosomes (C3 < 1 ≤ C2 ≤ 6 < C1).

Results for finite exchange rates (P_α,_ P_Ω_ > 0) between α and Ω are shown in the Fig. S2 (Supporting information). Under these conditions, the regulatory states of α- and Ω-cells approach each other, that is, the cells in the niche become indistinguishable from cells in the Ω-environment. Without exchange, the cells in the niche are isolated and clonal competition is active in the Ω-environment only. With exchange, competition extends to both environments and a mono-clonality conversion can be observed in the system in the long-term run. As the clones are identical, neutral competition is observed. Accordingly, the number of clones decreases with simulation time as 1/t. In all, following simulations exchange between the environments is assumed to take place (P_α_, P_Ω_ > 0).

Changes of the genomewide transcription, as those described, will change function of the cells, that is, will induce ARPs. In the following, we study the emergence of such ARPs in expanding cell populations and ask whether there is a feedback of the competition between young and aged cell clones on the distribution of regulatory states in these cell populations.

### DNA methylation controls the emergence of ARPs

**Figure 3:**
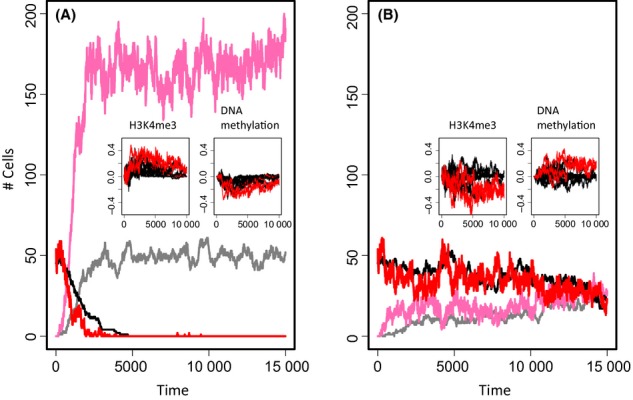
Age-related phenotype feedback on epigenetic states. (A) Simulated cell numbers for decreased differentiation rate *q* (D_NOVO_ = 0.3, TS = 2). Shown are cell numbers in α (black: young, gray: old) and in Ω (red: young, pink: old). (B) Simulated cell numbers for decreased proliferation rate *R* (D_MAIN_ = 0.8, TS = 2). Colors as in A. Inserts: Differences in histone and DNA methylation between systems without and with ARP. Changes in phenotype controlling genes (red) and other C1a-genes (black) are shown as averages over all cells of the system. (A) In case of a dominant ARP, aging of all C1a-genes becomes accelerated, that is, histone modification (DNA methylation) in the system without a phenotype is larger (smaller) compared to the system with an ARP. (B) In case of a recessive ARP, aging becomes selectively retarded in C1a-genes controlling the ARP but not in the other C1a-genes.

Typical phenomena associated with stem cell aging are a decrease in the potential to differentiate and to proliferate (Pollina & Brunet, 2011). These changes lead to competitive or recessive behavior of the aged clones, respectively. We asked whether epigenetic drifts within populations are affected by the resulting positive or negative selection of the aged cells and how expansion of the aged clones depends on the DNA-methylation machinery. We assumed that if the average transcription level of three selected C1a-genes (Fig. S5) falls below a threshold value (TS, see Table S1) either differentiation or proliferation becomes retarded. Figure 3 illustrates the consequences of these changes on the systems dynamics. More details are given in the Figs S3, S4 (Supporting information).

*A decreased differentiation rate q* (here *q* = q_0_/3, see Table S1) of the aged cells leads to an increase in the number of cells in the Ω environment upon occurrence of the ARP (Fig. 3A). Clones with aged cells overtake the system shortly after their occurrence (Fig. S3). Positive selection of the aged cells generates feedback on the cell’s regulatory states. In fact, it enforces silencing of all C1a-genes in aged cells (Fig. 3A, insert).

As C1a-genes are selected to control the ARP, fixation of the ARP requires stable silencing of these genes. Thus, for vanishing de novo methylation, the cells re-establish histone modification after replication and the genes associated with the respective nucleosomes show only a transient decrease in expression after cell division. Accordingly, the ARP cannot become dominant and only a few cells acquire it for a finite time (Fig. S4). The amount of such cells depends, for example, on the transcription state defining the phenotype and the ratio between the time scale required to re-establish the histone modification and that of cell replication (not shown).

*A decreased proliferation rate R* (here by *R* = 4 R_0_/5) of the aged cells enables the young phenotype to coexist with the aged one for long times (Fig. 3B). Over time, transcription approaches the threshold TS defining the aged phenotype in more and more cells. Accordingly, the frequency of adapting the phenotype increases, while the remaining fraction of young cells decreases. However, positive selection of young cells prevents complete dominance of the ARP (Fig. S3). In contrast to the case of dominant clones, the selection process affects only the transcription of the genes encoding the ARP. These genes remain expressed, while all other genes still underlie silencing (Fig. 3B, inserts).

Positive selection requires spontaneous fluctuations of the transcriptional state. Without fluctuations, switches between the phenotypes become impossible and the ARP becomes dominant. Fluctuation can be reduced, for example, by increasing maintenance of DNA methylation or changing the transcription threshold defining the ARP (Fig. S4). However, even for complete maintenance of DNA methylation, young cells can be observed in the long-term run because methylated C1a-genes are bistable and can spontaneously switch between high and low H3K4me3 states (Fig. S1).

These examples demonstrate that on one hand, aging can become accelerated if the ARP is dominant; on the other hand, silencing of genes encoding a recessive ARP is suppressed by clonal competition. In any case, the emergence of the ARP is sensitively modulated by the DNA methylation machinery. After this general analysis, we now apply the model to a specific stem cell system.

### The aged HSC phenotype activates young HSCs and retards overall aging

HSCs are probably those stem cells for which aging has been studied in most detail (Geiger *et al*., 2013). It is well known that HSCs populate niches where they stay most of the time quiescent. Occasionally, they leave the niche and start proliferation (Wilson *et al*., 2008). Recent studies demonstrated that aging of HSCs is associated with proliferation-dependent alterations of DNA methylation (Beerman *et al*., 2013), suggesting that aging actually requires activation of these cells in nice agreement with the assumption made in our model. So, we used the HSCs as model system for a first application of our multiscale model. It was our objective to reproduce experimental results on the competitive behavior of young and old HSC clones.

Recently, the competitive behavior of young and old HSCs transplanted into young mice has been studied applying ‘barcoding’ techniques (Verovskaya *et al*., 2013). It was demonstrated that after transplantation: (i) young cells more effectively contribute to the pool of differentiated cells but (ii) clones of aged cells are still competitive and do not die out within a time span of at least 20 weeks. A further phenomenon observed in mice and human is, that the number of HSCs increases with age (Dykstra *et al*., 2011; Pang *et al*., 2011). In mice, this effect is found to be more pronounced in the quiescent (CD34^−^) fraction of so-called ‘LSK HSCs.’ This suggests that in fact the number of HSCs in the niches becomes increased.

**Figure 4:**
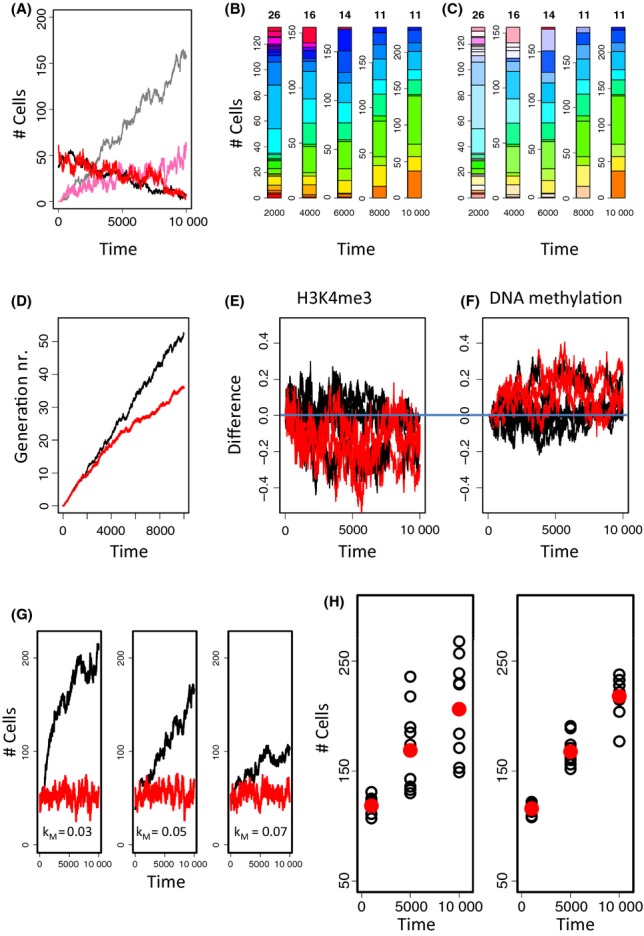
Simulated hematopoietic stem cell (HSC) aging. (A) The number of HSCs in the niche α increases with age, while the HSC number in Ω remains constant. Colors were chosen as in Fig. 3A. (B) Clonal composition of the system. Numbers at the top indicate the number of clones present in the system. Each clone is represented by an individual color. (C) The color saturation indicates the fraction of aged cells. Aged and young cells coexist for long times. (D) The average generation number is reduced in the HSC- (red) compared to the control system. (E,F) Differences in the epigenetic states in the HSC system compared to the system without an ARP. Phenotype controlling genes (red) and other C1a-genes (black) show a tendency for higher modification of the associated nucleosomes (E) and lower DNA methylation (F) in HSCs. (G) Emergence of the ARP sensitively depends on the histone modification rate k_M_. Decreasing k_M_ (left, 0.6 k_M__,0_) strongly accelerates aging, while increasing it (right, 1.4 k_M__,0_) decelerates it. (H) As a consequence, a high interindividual variance of the cell number is observed assuming individuals with differences in k_M_ (left), in contrast to a group of individuals with identical k_M_ (right). Black open symbols are results for 10 different individuals. Red dots are average values.

In particular, the latter finding cannot be explained by the scenarios described previously assuming changes of *R* or *q*, because these parameters do not influence the number of cells in the niche. This number will increase assuming either an increased inflow P_Ω_ or a decreased outflow P_α_ of cells (Material and Methods). Figure 4 shows simulation results on a system where the ARP is characterized by both an increased P_Ω_ (4.0-fold) and a decreased P_α_ (0.25-fold).

Under these assumptions, the number of aged cells increases roughly linearly with time, while that of the young cells decreases linearly (Fig. 4A). Thereby, in the long-term run, the number of cells in the niche approaches 4 times the number observed in the young system, while the number of cells in the Ω-environment remains unchanged. According to the changed distribution of the aged cells between α and Ω, they contribute less effectively to the pool of differentiated cells when calculated with respect to their total number. In the aging system, we found young and aged cells to coexist for more than 5.000 time steps (Fig. 4B,C) before the ARP overtakes the system. In the simulated HSC system, this refers to about 1 year (see: Table S1).

As these results are all consistent with the experimental findings mentioned previously, our simulations suggest that the ARP of HSCs retards aging of their population by the following mechanism: Due to their changed properties, aged cells tend to accumulate in the niche. This accumulation in turn increases the probability P_α_ of cells to leave the niche (Material and Methods). In particular, it leads to an increased flow of young HSCs out of the niche and thus activates them to start expanding, while allowing still for an effective accumulation of aged HSCs in the niche. This results in an optimized use of the stem cell resources, which can be seen on both the population and the molecular level. In fact, the average generation number reached in the simulated HSC system is smaller compared with a system without an ARP (Fig. 4D). Epigenetic silencing of age-related (C1a) genes is slowed down in the HSC scenario (Fig 4E,F).

In our simulations, we found that the emergence of the ARP of HSCs sensitively depends on the time ratio between cell proliferation and histone modification. Figure 4G shows that changes of the histone methylation rate by < 40% strongly affect the systems dynamics. Assuming such changes, our model simulations show a very large variance of the stem cell number in aged individuals (Fig. 4H). Thus, our results suggest that the huge inter-individual variance of the stem cell number with age as observed by Dykstra *et al*., (2011) might be due to moderate interindividual differences of the activity of histone methyltransferases.

## Discussion

Our model of stem cell aging links H3K4me3 modification and DNA methylation dynamics to describe age-related drifts in transcriptional states. The proposed aging process originates in the limited cellular capability to inherit epigenetic information. During cell replication, random distribution of modified nucleosomes on the daughter strands destabilizes the histone modification states. In case of methylation of H3K4, this destabilization opens time windows for methylation of the associated DNA that represses the histone states and enables long-term changes in the cell’s transcriptome. The process is general in that it runs in principal in all proliferating cells and at the same time specific as it depends on the original epigenetic state of the cells under consideration. The related transcriptional changes are potentially associated with ARPs. Here, we studied how these ARPs emerge and evolve depending on the stem cell environment.

Obviously, whether a particular ARP can be observed depends on its competitive properties, as for example, its proliferation and differentiation capacity. However, we found that the time scale required for its emergence and the degree to which it becomes fixed depends also on the properties of the histone and DNA modification machinery. Changes of these properties, as for example, of the DNA de novo methylation or the histone de-modification constant, can lead to fast activation, but also to repression of ARPs. Moreover, whether drifts in regulatory states occur depends also on the ratio of time scales, for example, of histone modification and cell replication. The model suggests that fast cell replication leads to progeroid phenotypes. This is a well-known phenomenon that has been analyzed already in a broader context (de Magalhaes & Faragher, 2008). We here provide a mechanistic explanation for this phenomenon.

As a particular application, we studied conditions under which our model system reproduces recent experimental findings on HSC aging. The obtained ARP is characterized by changed transition probabilities of the cells between the niche and the proliferative environment. Such an ARP has actually been observed (Florian *et al*., 2012). It originates in an increased expression of the Rho GTPase CDC42 that is known to induce quiescence and to improve homing of HSCs (Yang *et al*. 2007).

Strikingly, our model simulations suggest that both the young and the aged HSC phenotype use the niche as age-protective environment. In the young system, HSCs stay quiescent in the niche thus being largely protected from aging. In the aged system, old HSCs accumulate in the niche and push young cells out of it and accordingly ‘force’ them to age. This ‘homogenization’ results in a retardation of aging of the HSC population.

The increased residence times of aged HSCs in the niche partly enable them to re-establish lost histone modification states. Our model suggests that increased histone modification activity leading to a rapid reset of the histone marks will strengthen this kind of ‘rejuvenation’. Based on this mechanism, our model provides a possible explanation of the large interindividual variance observed in HSC numbers in mice (Dykstra *et al*., 2011). It suggests that a relevant part of this variance refers to interindividual differences in the activity of histone methyltransferases, which triggers different ‘aging rates’.

A prerequisite of the reactivation of the young phenotype in our model are sufficient fluctuations in the histone modification states. A strong limiting factor of such fluctuations is the maintenance of DNA methylation. Changes in this mechanism are thus predicted to largely affect ARPs. Actually, DNA de-methylation has been demonstrated to facilitate reprogramming of stem cells (Feng *et al*., 2009). As an alternative to DNA de-methylation, the model suggests that forced changes in the transcription of the genes controlling the ARP are able to improve state fluctuations and thus to facilitate rejuvenation. We like to note that rejuvenation of HSCs was actually observed by Florian *et al*., (2012) following CDC42 inhibition.

An open question in our model remains the exhaustion of the stem cell pool as observed in mice following serial bone marrow transplantation (Kamminga *et al*., 2005). A recent theoretical study by Glauche *et al*., (2011) suggested an age-related reduction of the capability of stem cells to regain regenerative potential in the stem cell niche. This phenomenological model somehow copes with our DNA-methylation scenario that blocks reversibility in histone modification and thus blocks reactivation of stem cell genes. By linking the decreasing regenerative potential to an effectively increasing differentiation rate, Glauche *et al*. were able to force the system to become exhausted. We expect similar results in our model coupling increasing DNA methylation of selected genes to increasing differentiation rates.

### Conclusions

We here suggest that the age-related functional decline observed in several stem cell systems is, at least in part, a consequence of the limited inheritance of epigenetic information and related progressive alterations of the transcriptome. We expect most of these changes to be deleterious. However, ARPs may have developed, as suggested here for HSCs, which function to maintain stem cell function by supporting mechanisms of stem cell rejuvenation. Understanding these mechanisms is a prerequisite of their potential utilization in stem cell therapies. Models of epigenetic stem cell aging like that presented here will support future research.

## Experimental procedures

The artificial genome (AG) model applied in this study is described in Fig. S5 (Supporting information). Transcription of the individual genes of this genome is calculated by solving:

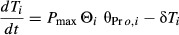
(1)

where δ is a degradation constant and P_max_ (=1000) the maximum promoter activity. Transcription activity of gene i is controlled by the occupancy of its promoter by polymerase II, θ_Pro,i_, which depends on the properties of the TF-network. Details about the regulatory principles, which are based on thermodynamics can be found in (Binder *et al*., 2010). In addition, transcriptional activity is assumed to be proportional to the binding probability Θ_i_ of the protein complex that incorporates the H3K4 histone methyltransferase to chromatin associated with gene i (see below).

We assume that the AG is wrapped around nucleosomes. The nucleosomes of subregions of the AG can act cooperatively regarding the modification of their histones. We here assume that these subregions are defined by the genes of the AG. Thus, all nucleosomes associated with a particular gene, but not nucleosomes from different genes act cooperatively regarding the modification of their histones. We assume one nucleosome per 200 bases of the AG.

To describe the histone modification process, we apply a model recently developed by Binder *et al*. (2013). A sketch of this model is shown in Fig. S6 (Supporting information). The chosen parameters are listed in Table. S1 (Supporting information). In general, the parameters have been chosen to emphasize the regulatory principles.

The model describes histone modifications assuming a positive feedback between the presence of the histone mark and the recruitment of modifying complexes as demonstrated for H3K4me3. The number of modified nucleosomes n_HM_ out of the N_H_ cooperative nucleosomes associated with the regulatory region of gene i is calculated by solving:

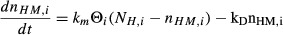
(2)

Here, k_D_ and k_M_ are the de-modification and modification rate, respectively. The binding probability Θ_i_ of the protein complex that incorporates the histone methyltransferase (see equation 1) is given by:

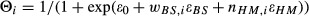
(3)ε_0_ is the ground enthalpy per bound complex, and ε_BS_ and ε_HM_ are the free enthalpy changes of DNA binding and H3K4me3 binding, respectively. We considered all un-methylated CpGs located in the regulatory region of gene i as potential binding sites (Thomson *et al*., 2010). We calculated the average free enthalpy change of DNA binding as (w_BS,i_ ε_BS_) Where w_BS,i_ is the probability of finding a nearby CpG un-methylated. Assuming independent methylation of all CpGs associated with the gene this probability is simply given by the fraction of un-methylated CpGs.

To account for changes of the DNA methylation, we applied the basic model of DNA methylation introduced by Sontag *et al*. (2006) which describes the parallel action of maintenance and de novo DNA methyltransferases (Smith & Meissner, 2013). In this Markov-chain model, CpGs can change their methylation states during cell division only. If a cell is dividing, maintenance methylation ensures that DNA methylation is conserved with a probability D_main_ smaller than one. Accordingly, not all CpGs keep their methylation status. In addition, DNA de novo methylation can occur with probability D_novo_ > 0 depending on the modification state of the associated nucleosomes. Recruitment of de novo methyltransferases has been shown to depend on the modification state of the associated histones. In fact, H3K4 methylation has been found to protect associated DNA from becoming de novo methylated. Accordingly, we assumed the de novo methylation rate D_novo_ of a CpG located in the regulatory region of gene i to depend on the modification of the nucleosomes associated with the gene:

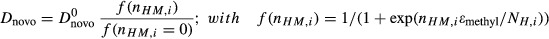
(4)

Here, *D*_novo_^0^ is the de novo DNA-methylation constant for CpGs located in a regulatory region of a gene, the histones associated to it are completely unmodified. ε_methyl_ is the interaction energy between the DNA methyltransferase and the modified nucleosomes associated with the gene.

In our model, we consider two environments, a niche environment α were cells are quiescent and a environment Ω, were stem cells can grow, divide and differentiate. In case, a single phenotype is present in the system, the probabilities for exchange between α and Ω, *P*_α_ and P_Ω,_ are further specified as following: *P*_α_ = ρ_α_*N*_α_ and *P*_Ω_ = ρ_Ω_/*N*_Ω_. *P*_α_ scales with N_α_; thus a high occupancy of the niche forces the cells to leave it. P_Ω_ inversely scales with N_Ω_, thus the probability of all cells from Ω to move to α (P_Ω_N_Ω_) is fixed, that is, there is a constant inflow as long as there is a cell in the Ω-environment. Cells residing in Ω proliferate with rate *R* and differentiate with rate *q* = q_0_ N_Ω_. Differentiated cells are eliminated from the system. The cell numbers in α and Ω change with time as:

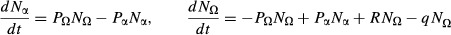
(5a,b)

Thus, in a stationary state (*dN*/*dt* = 0), one observes: *N*_α_ = (ε_Ω_/ε_α_)^1/2^ and *N*_Ω_ = *R*/q_0_. In case, two different phenotypes are present in the system (young, aged) one has to consider Equations (5a,b) for the different phenotypes in parallel as well as the transition probabilities between the phenotypes.

## Supporting Information

**Fig. S1** Stability of histone modification states. **Fig. S2** Stable phenotypes with cell exchange between α and Ω. **Fig. S3** ARP with changed proliferation and differentiation properties. **Fig. S4** Phenotypic changes are controlled by DNA-methylation. **Fig. S5** Artificial Genome Model. **Fig. S6** Transcriptional regulation by histone modification. **Table S1** Model parameters.
