# Supplementary material for: Understanding epigenetic changes in aging stem cells – a computational model approach
Source: Aging Cell. 2014 Jan 15;13(2):320–8. doi: 10.1111/acel.12177 (PMC4331773; doi:10.1111/acel.12177)
Supplement: Supplementary file 1 — Fig. S1 Stability of histone modification states. Fig. S2 Stable phenotypes with cell exchange between α and Ω. Fig. S3 ARP with changed proliferation and differentiation properties. Fig. S4 Phenotypic changes are controlled by DNA-methylation. Fig. S5 Artificial Genome Model. Fig. S6 Transcriptional regulation by histone modification. Table S1 Model parameters. [file acel0013-0320-sd1.docx]

**Supporting Information**

We here provide information about: i) the stability of histone modification states (Fig. S1), ii) the changes of the regulatory states in case of finite exchange rates between α and Ω (Fig. S2), iii) the impact of ARPs associated with retarded differentiation or proliferation on clonal competition (Fig. S3), as well as about iv) the impact of DNA methylation on the occurrence of these phenotypes (Fig. S4).

Moreover, we give a more detailed description of the artificial genome model (Fig. S5) and additional information on the histone modification model (Fig. S6).


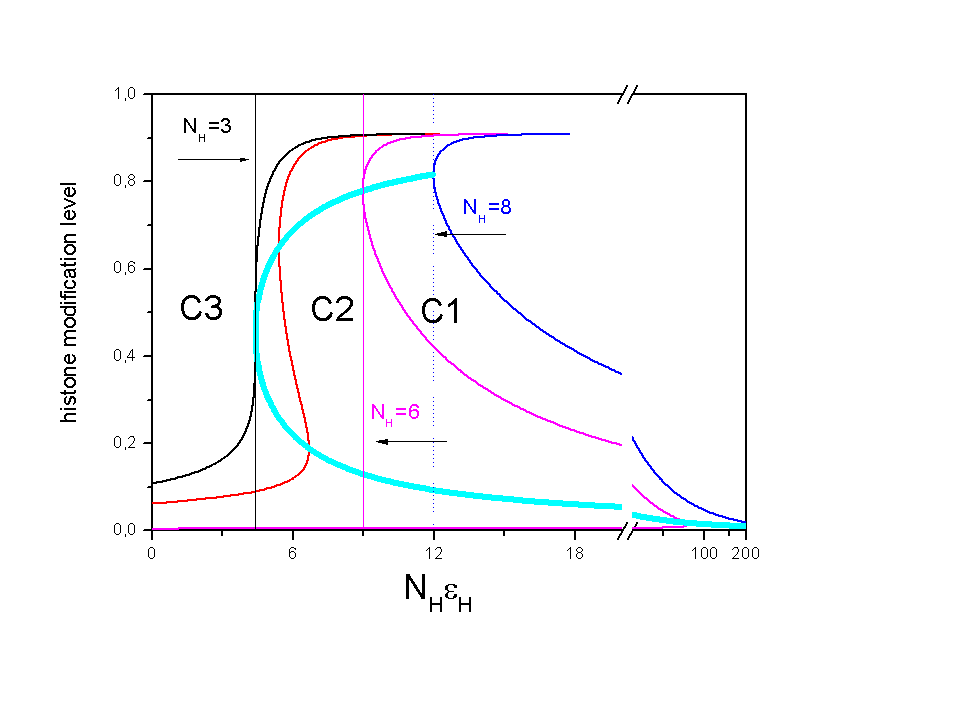


**Fig. S1: Stability of histone modification states.**  Shown are analytical solutions for the average fraction of modified nucleosomes in dependence of the maximum interaction energy of the modification complex with the modified nucleosomes N_H_ε_H_ in units of k_b_T; ε_H_=1.5 k_b_T (compare also (Binder *et al.* 2013)). The black, red, magenta and blue curve are solutions for 0%, 10%, 60% and 100% methylation of the associated CpGs. Between the intersections with the cyan line the solutions are instable, i.e. for the associated values of N_H_ε_H_ the system is bi-stable. We have chosen the parameters such, that without DNA methylation all histone modification states are mono-stable (black curve). Genes shorter than N_H_ ~3 (gene set C3) are associated with low modification states and thus are silent independent of DNA methylation. Genes above this threshold are associated with high modification states if the cells do never proliferate. DNA methylation in course of proliferation can render the latter genes, but only those with N_H_<6 (gene set C2), bistable and eventually monostable for a low histone modification state. The threshold N_H_=6 is defined by the equilibrium level of DNA methylation at vanishing histone modification, which is 60% for the chosen parameter sets (magenta line). It can approach N_H_=8 in case the equilibrium DNA methylation level approaches 100%. Genes longer than C2-genes (gene set C1) can spontaneously be de-modified during cell division. Whether and how fast they regenerate their histone modification state depends on the frequency of cell division, and the properties of the histone modification and the DNA methylation machinery (see text). Those genes which become silenced under given conditions define the subset of ageing genes C1a. In general genes with a length longer than N_H_ ~54 define a set of genes (C0, for maximum DM 60%) that is associated with a monostable high histone modification state. This length of genes was not reached in our AG.

**
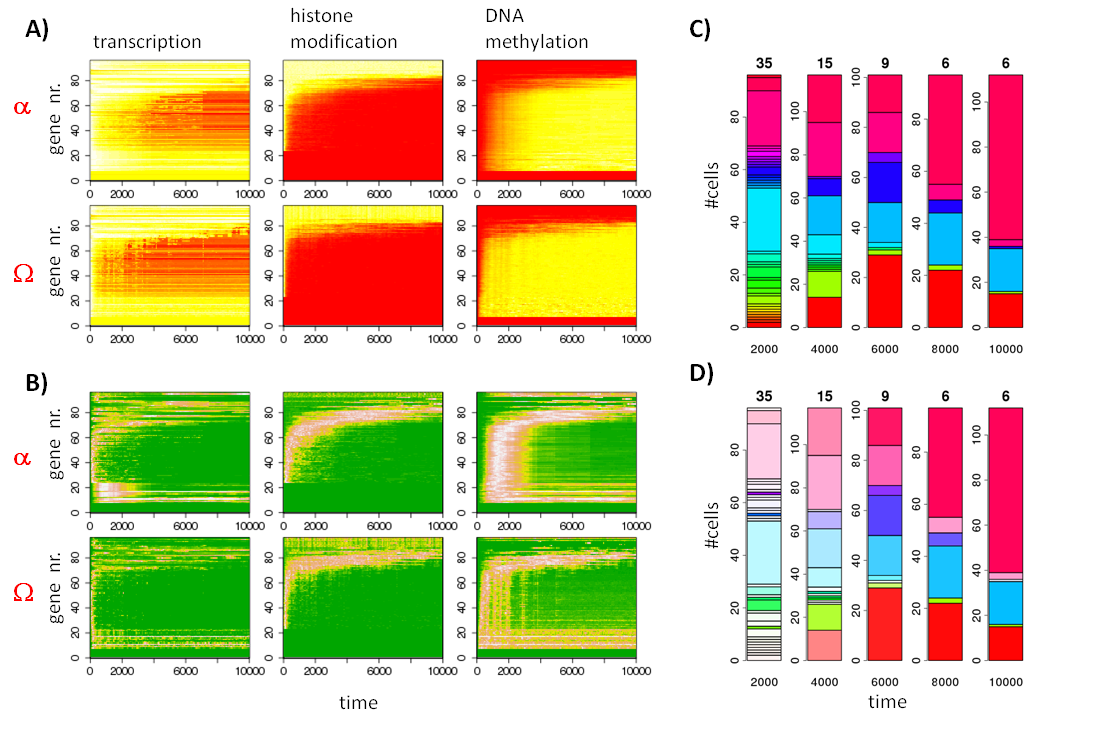
**

**Fig S2: Stable phenotypes with cell exchange between α and Ω.** A,B) Simulation results for the system shown in Fig. 2 but with a finite exchange of cells between α and Ω (colors as in Fig. 2). The regulatory states in the α- and Ω- environment approach each other at longer times. C,D) Results of the neutral clonal competition within the population. C) Time-dependent clonal composition. Numbers at top indicate the numbers of clones present in the system. D) Emergence of a pseudo ARP which is linked to the expression of the same gene set used in the ARP studies (TS=2) but is not linked to changes in phenotypic parameters.


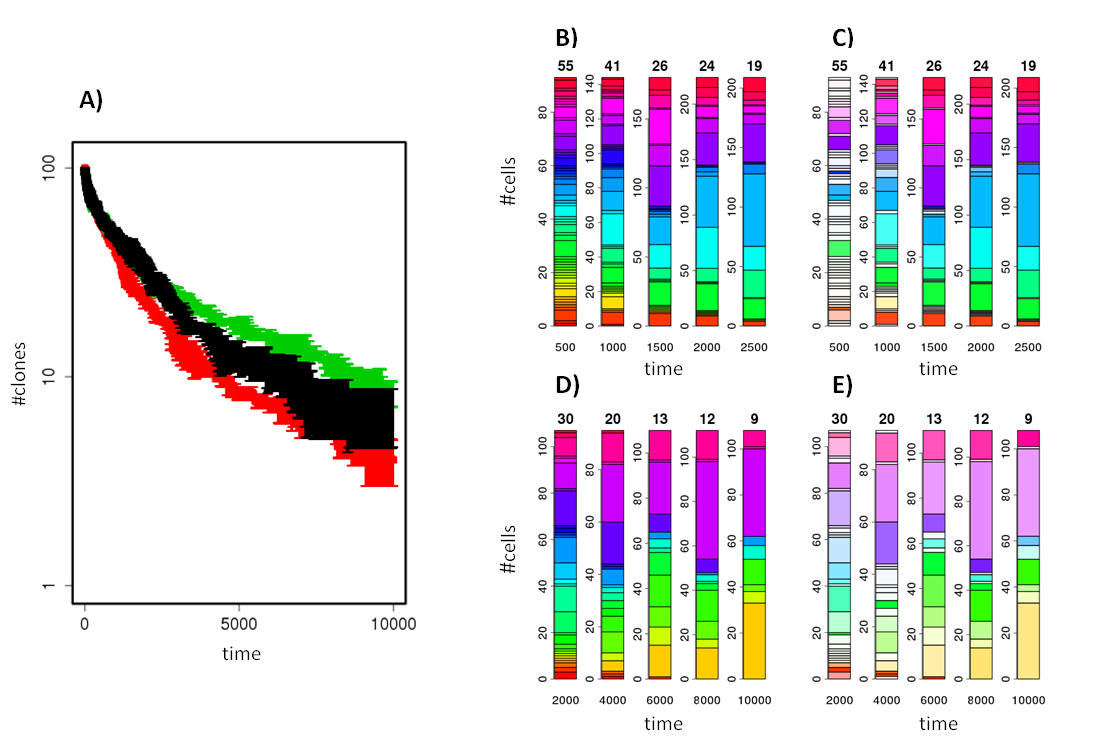


**Fig. S3: ARP with changed proliferation and differentiation properties.** Simulation results for the systems shown in Fig. 3A, B). A) Total number of clones in the system. An accelerated decrease compared to neutral competition (black line) is observed for retarded differentiation (red line) but not for retarded proliferation (green line). B-E) Clonal development within the system. Numbers at the top indicate the number of clones present in the system. B), D) Each clone is characterized by an individual color. C), E) Saturation of the colors shown in C), D), respectively is scaled with the fraction of aged cells in the clone. For retarded differentiation clones of aged cells rapidly overtake the system, while for retarded proliferation all clones age slowly.

**
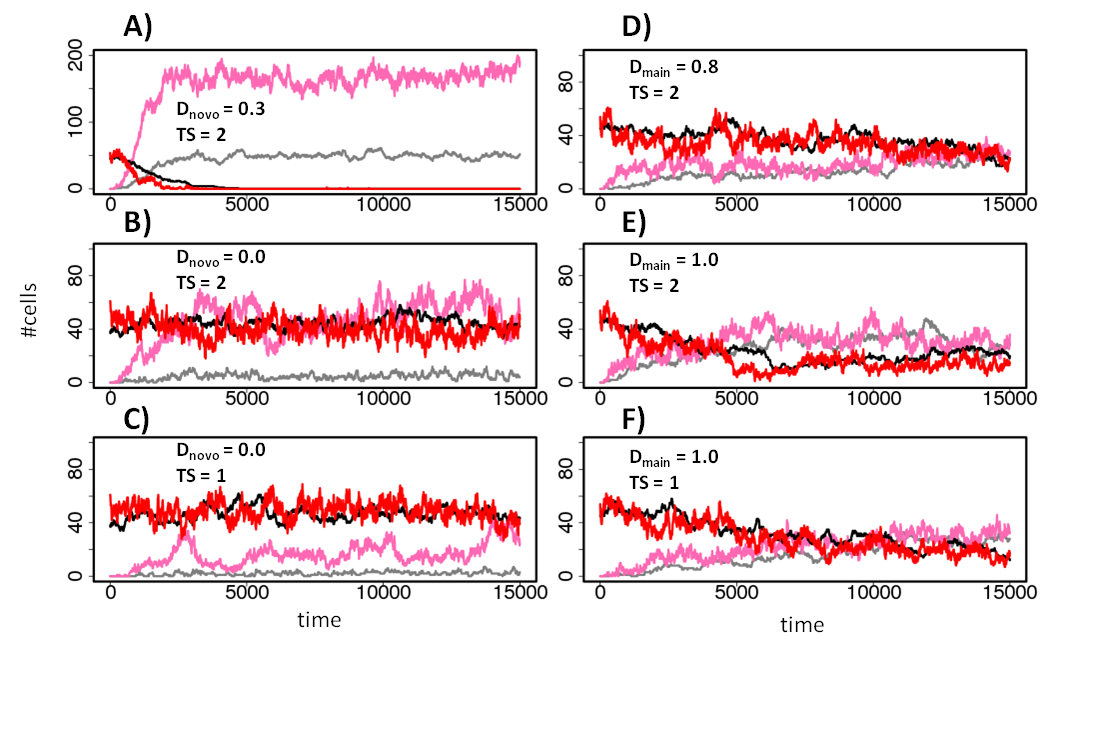
**

**Fig. S4: Phenotypic changes are controlled by DNA-methylation.** A-C) Simulated cell numbers for decreased q for different rates of de novo DNA methylation (D_NOVO_=0.3, 0.0, 0.0) and transcription thresholds (TS=2, 2, 1). Shown are cell numbers in α (black: young, grey: old) and in Ω (red: young, pink: old). For D_NOVO_=0.0 the dominant ARP does not overtake the system. Its appearance depends on the transcription threshold defining it. D-F) Simulated cell numbers for decreased R for different combinations of the probability of maintenance DNA methylation (D_MAIN_=0.8, 1.0, 1.0) and the transcription threshold (TS= 2, 2, 1). Colors as in A-C. For D_MAIN_=1.0 the recessive ARP largely overtakes the system. The dynamics depends on the transcription threshold TS defining the ARP.

**Defining the Artificial Genome**

The AG is constructed as a single strand genome by the following steps: 1) A string of length L_genome_ (=10^5^) consisting of four different digits (0,1,2,3) is generated. The digits are randomly chosen and equal distributed. Each digit corresponds to one letter of the (ATGC) genomic alphabet. 2) A promoter sequence of length L_prom_ (=5) digits is selected. Any position along the genome that matches this sequence is assumed to represent a promoter region of a gene. 3) The L_cod_ (=7) digits downstream the promoter define the coding sequence. All digits up-stream a promoter up to the next coding region define the regulatory region of the gene L_reg_. All together L_prom_, L_cod_ and L_reg_ constitute a gene (see Fig. S5).

Regulatory interaction between the genes is modeled as following: The coding sequence of a gene is translated into a binding sequence of the encoded TF by translating each number B of the coding sequence into a new number A of the binding sequence according to: A= B[mod4]; e.g. translates (3020202) into (0131313). Positions along the genome that match this binding sequence are considered as binding sites of the TF. TFs bound to the genome regulate the next gene downstream. We randomly assign each TF an activating or repressing function. These assumptions define a cis-regulatory (TF) network (Fig S5). The properties of this network can be directly calculated from L_genome,_ L_prom_, and L_cod_. In our simulations a realization with 96 genes was used.


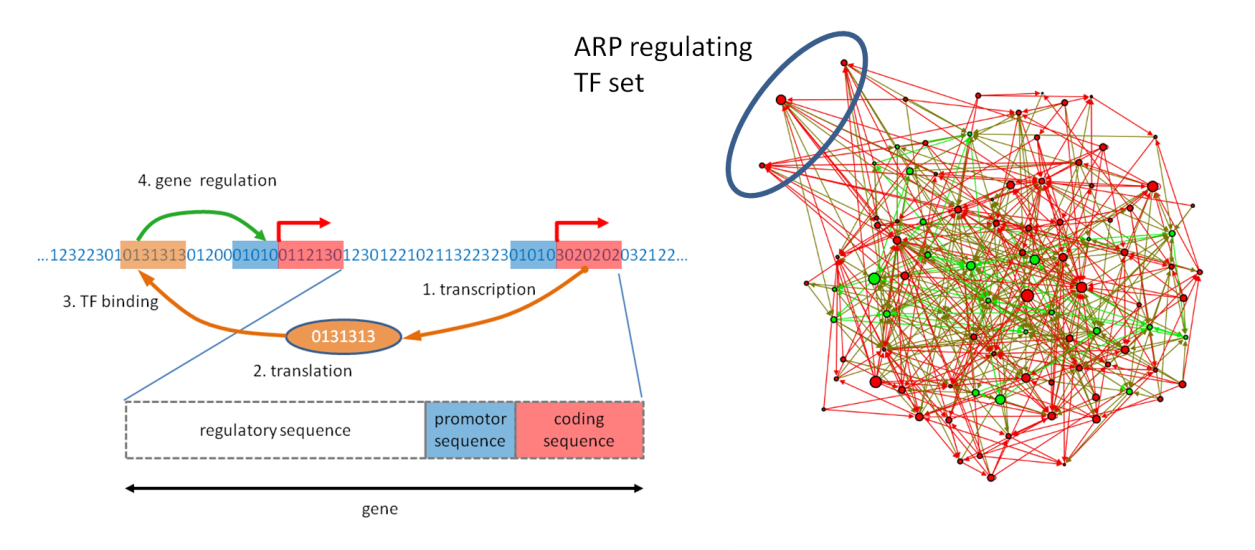


**Fig. S5 Artificial Genome Model.**  A string of numbers represents the AG sequence. The blue boxes contain the base promoter sequence (01010). Their occurrence divides the genome into different genes. Downstream each promoter the coding sequence of the gene is found (red box). These sequences are transcribed and translated into transcription factors (TF), which are (randomly chosen) either activators or repressors. TFs can bind the genome, at positions with equal sequence (orange box). Bounded TFs regulate the downstream gene (green arrow). B) TF network of the AG used in the simulations. Green nodes indicate activators and red nodes repressors. Lines describe interactions within the TF net. Their color refers to the type of the regulator (starting node). The subset of TFs encircled has been chosen to control the ARP. They are all encoded by C1a-genes.


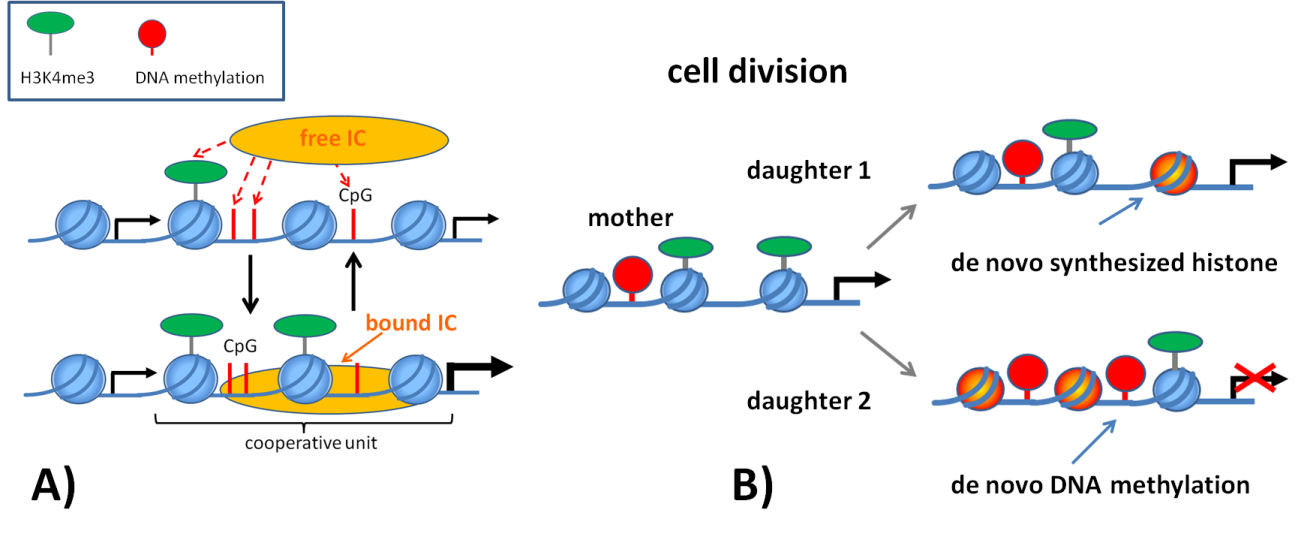


**Fig. S6: Transcriptional regulation by histone modification.**  A) Schematic plot of the histone modification model: Reversible binding of interaction complexes (ICs) to DNA is facilitated by interactions with specific binding sites (here unmethylated CpGs) and with H3K4me3 modified histones. Bound ICs catalyze histone modifications, giving rise to a positive feedback loop between IC-binding and histone modification. Transcription of the gene becomes activated after IC-binding. B) Epigenetic changes induced during cell division. Nucleosomes of the mother strand become randomly distributed onto the daughter strands and are complemented with de novo synthesized nucleosomes composed of unmodified histones (orange). DNA methylation of the mother strand is copied to the daughter strand by maintenance methylation; with finite probability only. De novo DNA methylation is possible at CpG not associated with H3K4me3 modified nucleosomes only.

**Tables**

| **Parameter** | **Symbols** | **Values** |
| --- | --- | --- |
| Ground enthalpy per bound interaction complex | ε_0_ | 10 k_b_T |
| Free enthalpy change of CpG binding | ε_BS_ | -5.5 k_b_T |
| Free enthalpy change of H3K4me3 binding | ε_HM_ | -1.5 k_b_T |
| Histone modification rate | k_M,0_ | 0.05 / Δt (±0.02 / Δt in HSCs) |
| Histone de-modification rate | k_D_ | 0.005 / Δt |
| DNA maintenance methylation probability | D_main_ | 0.8-1.0 |
| DNA de novo methylation probability | $D_{novo}^{0}$ | 0.0-0.3 |
| growth rate (after 10 growth steps the cell gets divided) | R_0_ | 0.1 / Δt  4R_0_/5= 0.08 Δt |
| Differentiation rate | q_0_ | 0.0000625 / Δt  q_0_/3= 0.0000208 / Δt |
| Exchange rate α 🡪 Ω | ρ_α_ | 0.00002 / Δt  HSC: 0.000005 / Δt |
| Exchange rate Ω 🡪 α | ρ_Ω_ | 0.05 / Δt  HSC: 0.2 / Δt |
| Interaction energy between DNA- and histone- methyltransferase | ε_methyl_ | 6 k_b_T |
| Transcription degradation rate | δ | 0.1 / Δt |
| Transcription threshold | TS | 1, 2 |

**Table S1: Model parameters.** The unit k_b_T refers to the Boltzmann unit, while Δt refers to simulation time steps. Thereby, 250 Δt refer to about 1 generation in the HSC system, which was suggested to refer to 18 days (Takizawa *et al.* 2011). Accordingly, 10.000 Δt refer to about 2 years. Parameters of the AG and the TF network were chosen as in (Binder *et al.* 2010). The energy constants were chosen from realistic ranges for protein binding of some k_b_T.
